# Germanium-Based Optical Coatings for Aesthetic Enhancement with Low Radiofrequency Attenuation

**DOI:** 10.3390/nano14060530

**Published:** 2024-03-15

**Authors:** Enrique Carretero, Rocío Chueca, Rafael Alonso

**Affiliations:** 1 Departamento de Física Aplicada, Universidad de Zaragoza, C/Pedro Cerbuna, 12, 50009 Zaragoza, Spain; ralonso@unizar.es; 2 Departamento de Ingeniería Electrónica y Comunicaciones, Universidad de Zaragoza, C/Maria de Luna, 1, 50018 Zaragoza, Spain; rchueca@unizar.es

**Keywords:** optical coatings, germanium, thin film, radiofrequency, sputtering

## Abstract

This work focused on developing optical coatings for decorative applications that remain transparent in the radiofrequency range. To achieve this, a combination of dielectric material (silicon-aluminum nitride, SiAlN_x_) and low-electrical-conductivity semiconductor material (germanium) was utilized. Germanium plays a crucial role in providing absorption in the visible spectrum, facilitating the design of coatings with various aesthetic appearances, while allowing for control over their transmittance. The optical properties of thin germanium layers were thoroughly characterized and leveraged to create multilayer designs with diverse aesthetic features. Different multilayer structures were designed, fabricated, and optically characterized, resulting in coatings with metallic gray, black, or various colors in reflection, while retaining the ability to transmit visible light for illumination and signaling applications. Finally, the radiofrequency attenuation of the developed coatings was measured, revealing negligible attenuation; this is in stark contrast to the metallic coatings used for decorative purposes, which can attenuate by up to 30 dB.

## 1. Introduction

The use of coatings for aesthetic purposes is a widely studied field with many applications. These coatings can be applied through various techniques, such as painting [1,2], electrochemical deposition [3,4,5], chemical vapor deposition (CVD) [6], and physical vapor deposition (PVD) [7,8,9,10], among others. PVD techniques are a commonly employed choice across different industries for aesthetic enhancement, such as metalizing components, especially in the automotive sector [11,12], or applying them to building glazings [13].

PVD techniques, particularly magnetron sputtering, enable the deposition of thin film coatings composed of a wide array of materials, while providing precise control over the thickness of these layers. This capability allows for the fabrication of interference optical coatings, achieving an aesthetic appearance and optical properties that are unattainable with simple paints [14,15]. Moreover, the spectral transmittance of the coatings can be meticulously controlled. Interference optical coatings enhance the optical properties of surfaces and are extensively applied in the production of antireflective coatings, optical filters, low-emissivity glasses, and more [16,17,18,19].

The calculation used to determine the optical properties of such coatings is well established, with several models available for this purpose [18,20,21]. Open access programs also exist, facilitating both the calculation of these properties and the determination of optimal layer thicknesses based on a given target spectrum [22].

The fabrication of dichroic optical filters, employing dielectric materials, allows for the attainment of various spectral curves, with the constraint that they exhibit no optical absorption. Consequently, the sum of the transmittance and reflectance values equals one for all wavelengths. However, when the goal is to develop coatings with optical absorption, aiming to achieve relatively independent transmittance and reflectance, the use of materials with optical absorption is necessary [23,24]. In other words, these are materials with an imaginary component in their refractive index. This can be easily accomplished through the utilization of metallic materials; nevertheless, the high electrical conductivity of these materials results in the high reflectance (and absorption) of electromagnetic waves in the radiofrequency range [25,26].

A parameter tightly linked to the absorption, transmission, and reflection of radiofrequency in thin films is sheet resistance. In [27], expressions for transmitted amplitudes are calculated based on the various physical parameters of thin films, such as thickness and conductivity. From the study of these expressions, it was deduced that, to achieve high-level radiofrequency transmission, it is necessary for R_s_ to be significantly greater than Z_0_, where R_s_ represents sheet resistance and Z_0_ is the impedance of a vacuum (377 Ω). Conversely, when R_s_ is much lower than Z_0_, the coating will exhibit high radiofrequency attenuation.

In the current landscape, the significance of electromagnetic waves in the radiofrequency range has surged, given the widespread use of mobile phones that operate across various radiofrequency bands, spanning from 0.7 to 3.6 GHz. Consequently, when considering coatings for construction applications, it is crucial for them to exhibit less attenuation within these radiofrequency bands. This consideration has gained particular prominence in the domain of low-emissivity coatings, prompting the development of solutions that make use of frequency-selective surfaces (FSS) [28,29]. In the automotive industry, the development of decorative coatings with minimal radiofrequency attenuation is also a noteworthy pursuit, driven by the current utilization of radar systems in vehicles.

This study focuses on the development of coatings with various aesthetic appearances using germanium as a material with optical absorption. Germanium, being a semiconductor material with a bandgap energy of 0.66 eV, exhibits absorption in the visible spectrum, but has less conductivity compared to that of metals. This characteristic enables the creation of coatings with low radiofrequency attenuation, while achieving diverse aesthetic effects, such as black coatings, metallic gray coatings, or coatings in different colors [30,31]. Moreover, these coatings may have semi-transparency in the visible spectrum, and multilayer structures are devised to modulate the visible transmittance factor of the coating.

## 2. Materials and Methods

The samples were deposited using DC pulsed magnetron sputtering in a semi-industrial facility, where the samples moved linearly facing the target. The target sizes were 600 mm × 100 mm, and the large size of the materials combined with their dynamic deposition allowed for the production of coatings with homogeneous and uniform thickness on substrates measuring up to 520 mm × 300 mm. ClearTrans ceramic glass substrates from Schott were used, which were cleaned with ACEDET detergent prior to the deposition process. Additionally, a treatment to clean and activate the surface was conducted using an Ar+ ion beam generated by introducing a flow of 50 sccm (standard cubic centimeters per minute) of Ar into the process chamber and applying an accelerating voltage of 2 kV.

The materials employed included 99.99% pure germanium (Ge) and a silicon (90%)/aluminum (10%) alloy with a purity of 99.99%. The deposition process took place under a base pressure below 10^−4^ Pa and a working pressure on the order of 10^−1^ Pa. The deposition of Ge layers involved introducing a flow of 200 sccm of Argon into the chamber (corresponding to a pressure on the order of 10^−1^ Pa) and applying a power of 700 W (1.17 W/cm^2^). The deposition of SiAlN_x_ layers was carried out using reactive sputtering, introducing a flow of 100 sccm of Argon and 100 sccm of N_2_. A power of 2.5 kW (4.17 W/cm^2^) was applied during this process.

The deposition rate and layer thickness were calibrated using a Dektak XT (Bruker, Billerica, MA, USA) mechanical profilometer. The layers of each material (Ge and SiAlN_x_) were individually deposited under the same deposition conditions as those of the decorative coatings. However, a slower substrate holder speed was employed, resulting in a longer sample deposition time, aimed at achieving layers of approximately 100 nm thickness and enhancing the thickness calibration precision. Subsequently, for the deposition of the decorative coatings, the substrate holder speed was adjusted to achieve the desired thickness for each coating layer. This adjustment followed an inverse proportionality rule, wherein a faster substrate holder speed corresponded to a shorter deposition time and thinner deposited layer.

Spectrophotometric measurements were conducted using a homemade UV-VIS-IR spectrophotometer, which measures both transmittance and reflectance at an incidence angle of 8° across the range from 300 to 2500 nm.

The complex refractive indices of the materials were calculated from the spectrophotometric measurements of transmittance and reflectance (from both sides) of the samples with only a deposited layer of approximately 30–50 nm. To determine the refractive indices of this layer, its thickness was measured, and a simple model of a layer on a substrate with a known thickness and refractive index was built. The system was numerically simulated, calculating each pair of values (*n* is the real part of the refractive index, and *κ* is the imaginary part), and an attempt was made to minimize the defined merit function *χ* as follows:

(1) $$\chi \left(n,\kappa \right)={\left(T_{calc}-T_{\exp}\right)}^{2}+{\left(Rc_{calc}-Rc_{\exp}\right)}^{2}+{\left(R_{calc}-R_{\exp}\right)}^{2}$$

where *T_calc_*, *Rc_calc_*, and *R_calc_* represent the theoretically calculated transmittance, coating-side reflectance, and substrate-side reflectance, respectively, for each value of *n* and *κ*. This calculation was performed using a single-layer model of Air/Substrate/Film/Air utilizing a known substrate (its refractive indices) and taking into account the generated interferences. Meanwhile, *T_exp_*, *Rc_exp_*, and *R_exp_* denote the experimentally measured values for the characterized sample. Merit function minimization was performed at each wavelength *λ*, thereby adjusting the values of the complex refractive index for all the spectrophotometrically measured wavelengths. From the imaginary part of the refractive index, the absorption coefficient *α* was calculated as follows:

(2) $$\alpha =\frac{4\pi \kappa }{\lambda }$$

The absorption coefficient of germanium calculated for different wavelengths was used to determine the bandgap energy through the Tauc plot [32], employing the following expression:

(3) $${\left(\alpha h\nu \right)}^{r}=A\left(h\nu -E_{g}\right)$$

where *r* represents a value depending on the type of transition, with *r* = 1/2 denoting indirect transitions. This method takes into account multiple reflections, resulting in more accurate results [33].

The optical properties were theoretically adjusted using proprietary software based on the continuity of tangential components of the electric and magnetic fields at the interfaces of the multilayer structure [20].

The transmittance and reflectance spectra measured with a spectrophotometer were used to calculate the values of visible transmission *T_VIS_* and visible reflection *R_VIS_* using the following expressions:

(4) $$T_{VIS}=\frac{\int_{0}^{\infty }T\left(\lambda \right)D_{65}\left(\lambda \right)V\left(\lambda \right)d\lambda }{\int_{0}^{\infty }D_{65}\left(\lambda \right)V\left(\lambda \right)d\lambda }$$

(5) $$R_{VIS}=\frac{\int_{0}^{\infty }R\left(\lambda \right)D_{65}\left(\lambda \right)V\left(\lambda \right)d\lambda }{\int_{0}^{\infty }D_{65}\left(\lambda \right)V\left(\lambda \right)d\lambda }$$

where *T*(*λ*)** represents spectral transmittance, *R*(*λ*)** represents spectral reflectance, *λ* represents the wavelength, *D_6_*_5_ represents the D65 illuminant defined by the International Commission on Illumination, and *V*(*λ*)** represents the sensitivity of the human eye. This calculation was performed following the EN 410 standard [34]. For color calculation, CIELab color coordinates were utilized, distinguishing between the color coordinates in transmission *LAB_Trans_* and reflection *LAB_Reflec_*.

Structural and morphological characterizations were performed using XRD with an RIGAKU device model D/max 2500 (Tokio, Japan) and using Field-Emission Scanning Electron Microscopy (FESEM) with a Carl Zeiss MERLIN model (Oberkochen, Germany).

**Figure 1:**
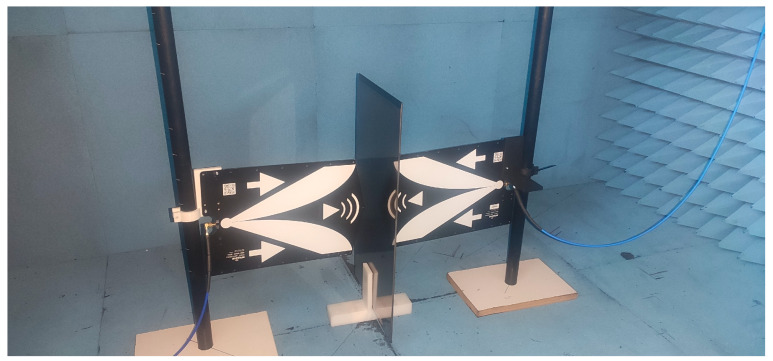
RF measurements setup.

The experimental setup for RF measurements (Figure 1) used two directional Vivaldi antennas (TSA600, RFSPACE, Atlanta, GA, USA) operating within the 600–6000 MHz frequency range and placed in an anechoic chamber. Signal generation and measurements were carried out using a vector network analyzer (picoVNA 106, Pico Technology, Cambridge, UK).

For the characterization of the different glass samples, a first measurement was made without placing anything between the antennas, which was taken as a reference. This was used to discount the effect of the cables, antennas, or secondary reflections on the anechoic chamber. Then, the glass sample was placed between the antennas, and a second measurement was taken. Attenuation was calculated as the difference between both these measurements.

## 3. Results and Discussion

### 3.1. XRD, FESEM, and Optical Properties of Ge

The coatings developed consisted of multilayers formed of SiAlN_x_ and Ge. SiAlN_x_ is a transparent dielectric material with low optical absorption values in the visible and near-infrared spectra, which is very similar to Si_3_N_4_, but with a slightly higher refractive index of approximately 2.08 at 550 nm compared to the value of 2.05 corresponding to Si_3_N_4_ [35].

**Figure 2:**
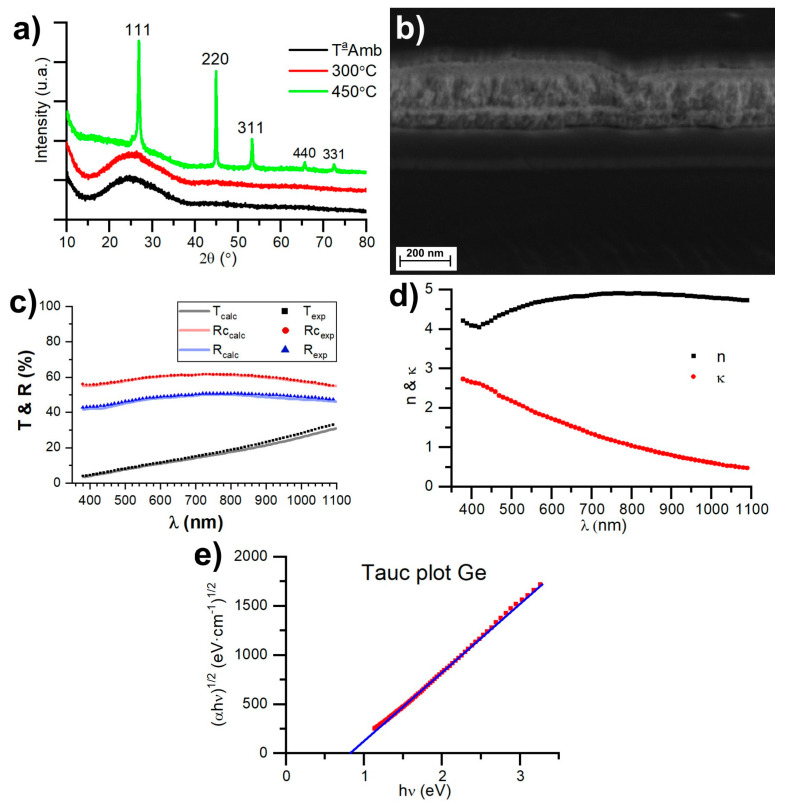
(**a**) XRD of Ge thin films; (**b**) FESEM image of five-layer coating made of Ge and SiAlN_x_; (**c**) spectrophotometric measurements of a 28 nm thin film of Ge and its calculated fitting using the found complex refractive indices; (**d**) complex refractive index of Ge thin films; and (**e**) Tauc plot of amorphous Ge thin films.

The optical properties of Ge play a crucial role in decorative coatings, as it is a material that absorbs light and can be used to modulate the reflectance and transmittance of coatings. Ge deposited at room temperature has an amorphous structure; if it is deposited with a substrate at 300 °C, it also results in an amorphous structure, while heating Ge to 450 °C leads to a crystalline structure (Figure 2a). Figure 2b shows an FESEM image of a multilayer consisting of five layers: glass/SiAlN_x_ (40 nm)/Ge (9 nm)/SiAlN_x_ (110 nm)/Ge (30 nm)/SiAlN_x_ (42 nm). Germanium exhibits a more solid morphology, while SiAlN_x_ is columnar.

Figure 2c illustrates the transmittance and reflection spectra (from both sides) of a 28 nm thin film of amorphous germanium. These measurements were employed to compute the complex refractive indices of amorphous germanium by minimizing the merit function χ, as defined in Equation (1). Additionally, the simulated values for transmittance and reflection are presented for a 28 nm thin film of germanium, utilizing the complex refractive index values obtained with this method.

Figure 2d displays the refractive indices obtained for amorphous germanium, which are similar to those obtained in [36], while the bandgap energy is E_g_ = 0.82 eV, which was calculated using a Tauc plot [32] (Figure 2e). An important aspect to consider is that these optical properties of germanium will remain intact as long as it remains amorphous. Therefore, all the designs for decorative coatings must be maintained at a temperature below 300 °C to ensure the amorphous structure of the germanium layers.

**Figure 3:**
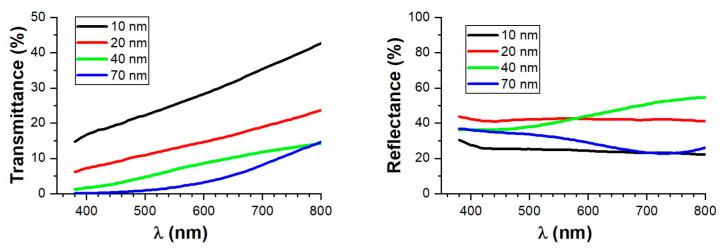
Simulated spectra of transmittance (**left**) and reflectance (**right**) for thin films with varying thicknesses of amorphous germanium deposited on 4 mm glass. The reflectance was simulated from the substrate side.

Once the complex refractive indices were obtained, we simulated the optical properties of thin films of amorphous germanium with different thicknesses, as shown in Figure 3. We can observe how the transmittance of the thin germanium layers increases with the wavelength, meaning that they transmit blue colors to a lesser extent than red ones. Consequently, simple structures containing germanium will appear reddish during transmission. This is an expected result since the imaginary part of the refractive index (related to absorption) increases as the wavelength decreases. Additionally, in Figure 3, we can also observe the reflectance spectra (from the substrate side) for the germanium samples of different thicknesses. For the thin ones, the reflectance increases uniformly for all the wavelengths until it reaches a value of 40% for the 20 nm thick layer. As we continue to increase the thickness of the layer, it stabilizes around 37% between 400 and 500 nm, while for longer wavelengths, there are certain “oscillations” in the reflectance values. This is because the reduced absorbance of germanium at these wavelengths allows for the interference of the reflected amplitudes at both interfaces of the germanium layer. Thus, the reflectance values are high when this interference is constructive and low when it is destructive. This effect was also observed to a lesser extent in the transmittance curves. For example, the 70 nm thick layer has the same transmittance as the 40 nm thick layer for a wavelength of 800 nm. This is because, in the 70 nm thick germanium layer, the interference produced is constructive during transmittance but destructive during reflectance (hence, it has lower reflectance than the 40 nm thick layer does).

### 3.2. Decorative Coatings

**Figure 4:**
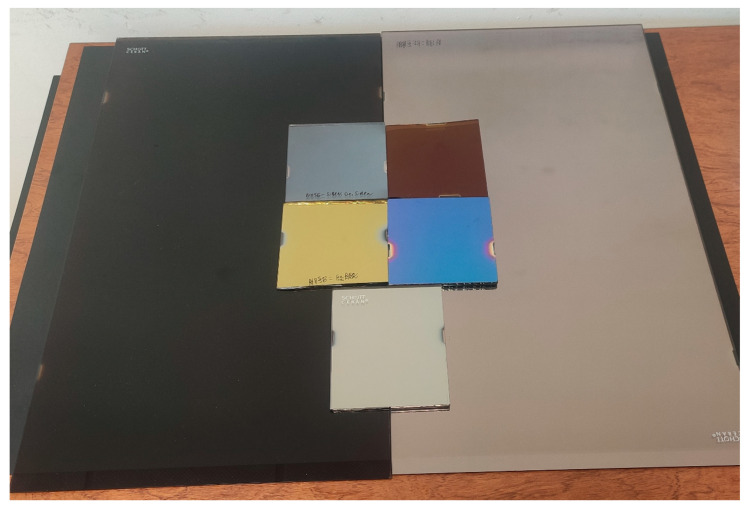
Optical coatings with different aesthetic aspects.

Decorative coatings with various aesthetic appearances have been developed. These coatings are categorized into three types: metallic gray, black, and colored ones. All these decorative coatings have been fabricated using alternating thin layers of SiAlN_x_ and Ge, precisely adjusting the thickness of each layer to control their optical properties. Precision is crucial because these properties are determined by the optical interference resulting from the multilayer structure. Figure 4 displays an image featuring different deposited coatings, including two large samples measuring 520 mm × 300 mm (Black and metallic gray) and several smaller ones measuring 100 mm × 100 mm.

#### 3.2.1. Gray Metallic

The metallic gray coatings are of great decorative interest. Two types of structures have been developed, resulting in a metallic gray color made of germanium, as shown in Table 1.

**Figure 5:**
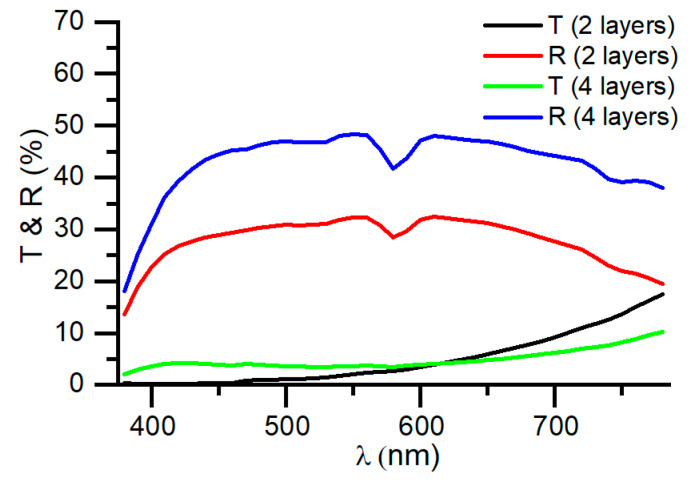
Transmittance and reflectance of two-layer and four-layer gray metallic coatings.

The two-layer structure consists of a first layer of germanium, providing a metallic appearance to the coating and allowing for the modulation of transmittance based on the deposited thickness of germanium. The second SiAlN_x_ layer is deposited to protect the germanium layer and optimize the visible transmittance of the coating, as well as strike a balance between the blue and red colors. Improved optical properties of a metallic gray coating can be achieved with a four-layer structure, alternating the Ge and SiAlN_x_ layers, as shown in Table 1. The transmittance and reflectance spectra of both multilayers are depicted in Figure 5, and the overall values of the optical properties are presented in Table 2. It can be observed that with the two-layer structure containing a single germanium layer, the achieved reflectance is 31%, whereas with four layers containing two germanium layers, a reflectance of 46% can be reached, resulting in a brighter metallic appearance that is similar to that of metal. In Table 2, it can be noted that chromatic coordinates a and b in reflection are very close to 0, implying the reflection of a neutral color.

The global value of visible transmittance *T_VIS_* can be controlled in both the structures by adjusting the thickness of the germanium layers. Another aspect that represents a significant advantage of the four-layer structure is that the transmittance curve is flat and remains approximately constant throughout the entire visible spectral range. This can also be observed in Table 2 by comparing the values of a and b in the color coordinates related to transmission. While *a* = 10.5 and *b* = 23.4 for the two-layer structure, in the four-layer structure, the values of *a* and *b* are very close to 0. This means that the transmitted color is neutral, and from the spectral transmittance curve, it is evident that it equally transmits all the wavelengths in the visible zone. This can be advantageous if lighting and signaling elements are to be used alongside these types of semi-transparent coatings, proving a clear advantage for the four-layer structure.

The transmittance curve of the two-layer coating is determined by the increased absorption of germanium at short wavelengths (blue), whereas in the four-layer structure, interference is generated to compensate for this increased absorbance of germanium at such wavelengths. In this context, the coating based on a four-layer structure is a type of Fabry–Perot interferometer [37,38], where the 80 nm SiAlN_x_ layer acts as a cavity, and the surrounding germanium layers serve as reflective surfaces. This configuration allows for the optimization of the thickness of the layers to increase transmittance in the desired spectral region. While more complex structures with up to three layers of germanium could be useful in some cases, in most instances, they would render the coating opaque. This is because the germanium layers in the four-layer coating are already very thin at around 20 nm; thus, it is challenging to make them thinner without indirectly affecting the transmittance or reflectance spectrum. Therefore, while adding a third layer of germanium would offer more design flexibility, it would also introduce optical absorption due to the increased total thickness of germanium. Consequently, we do not consider this to be a viable option for designing metallic gray coatings that are semi-transparent.

#### 3.2.2. Black Coating

Black coatings have also proven to be very interesting for industrial applications. In order to minimize the reflectance of the coating, a five-layer structure glass/SiAlN_x_/Ge/SiAlN_x_/Ge/SiAlN_x_ was developed, where by properly adjusting the thicknesses, the desired aesthetic appearance can be achieved. Specifically, it is crucial to adjust the thicknesses of the first three layers, which determine the aesthetic appearance of the coating, while the fourth layer (the second layer of germanium) is used to adjust the transmittance of the coating, and the final SiAlN_x_ layer is used to protect the coating and optimize the transmittance curve chromatically. Three different designs of the black coatings were produced, as shown in Table 3.

**Figure 6:**
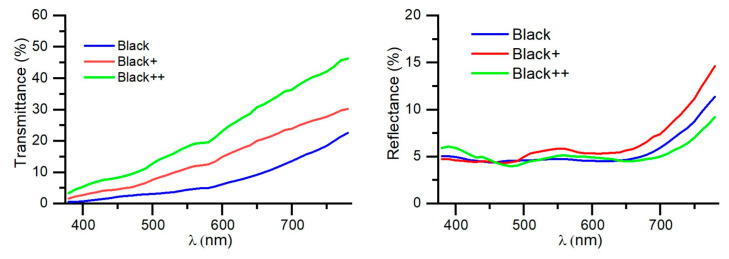
Transmittance (**left**) and reflectance (**right**) spectra of black coatings.

In Figure 6, the transmittance and reflectance spectra of the three designs are presented. It can be observed that the reflectance values of all three designs are very similar, around 4.5–5.5%. In fact, these reflectance values are primarily determined by the first surface of glass substrate, which is approximately 4%. The reflectance of the coating itself is less than 1%. The chromaticity of this reflectance is very neutral, as shown in the chromaticity coordinates indicating reflectance in Table 4.

On the other hand, the main difference among the three designs lies in their transmittance. While the black coating has a transmittance of 4.7%, Black+ has a transmittance of 11.6%, and Black++ has a transmittance of 18.6%. This demonstrates that this value can be controlled by adjusting the fourth layer of the structure (the second layer of germanium).

The chromaticity values of transmittance are similar in all cases due to the greater absorption of germanium at shorter wavelengths. This chromaticity of transmission could be improved with a seven-layer structure (adding a layer of Ge and another of SiAlN_x_) and adjusting the thicknesses so that interference can enhance the transmittance at short wavelengths in a manner analogous to that of the four-layer metallic gray coating.

#### 3.2.3. Colored Coatings

Coatings with different colors have been developed using a similar structure to the black coatings glass/SiAlN_x_/Ge/SiAlN_x_/Ge/SiAlN_x_ for blue and yellow, and a three-layer structure glass/SiAlN_x_/Ge/SiAlN_x_ was used for light blue. With the same five-layer structure as that of the black coatings, different colors can be achieved due to the versatility of the layer structure. Table 5 displays the thicknesses of each layer for blue, light blue, and yellow coatings.

**Figure 7:**
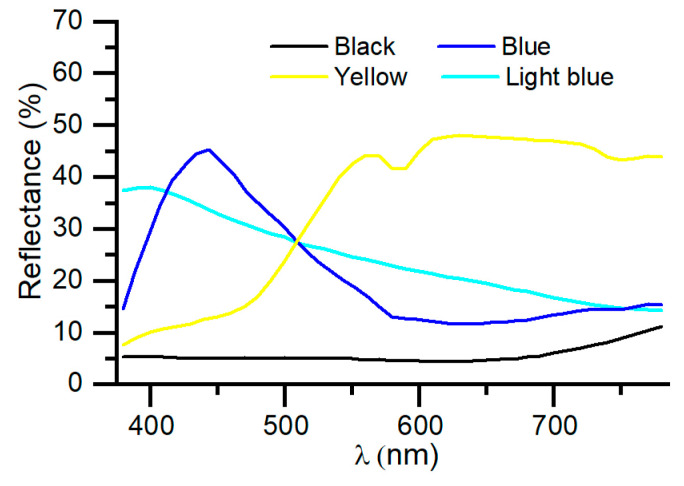
Reflectance spectra of colored coatings.

In Figure 7, the reflectance spectra of the blue, light blue, and yellow coatings are shown. The coating can be adjusted to obtain different aesthetic appearances. The overall values of transmittance and reflectance, as well as the color coordinates of these coatings, are shown in Table 6.

### 3.3. RF Measurements

**Figure 8:**
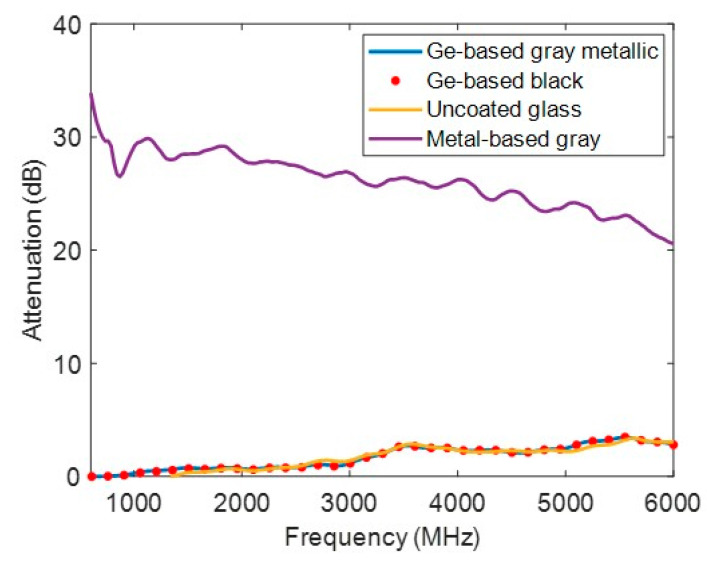
Radiofrequency attenuation of uncoated glass, gray and black (Ge-based one), and metallic coatings (metal-based one).

Radiofrequency attenuation is illustrated in Figure 8. Four samples were measured: uncoated glass, Ge-based gray metallic, Ge-based black, and metallic coatings (metal-based one). The results indicate that the attenuation of the germanium-based coatings developed in this study is comparable to that of the uncoated glass. Conversely, a metallic coating with 10 nm of silver can attenuate up to 30 dB more than uncoated glass can. This demonstrates the high-level radiofrequency transparency of the germanium-based coatings developed in this study, offering a significant advantage over the decorative coatings using conductive metals. These results align with the expectations of the classical electromagnetism theory applied to thin conductive layers [27]. Coatings made of germanium exhibit significantly more sheet resistance than the impedance of a vacuum, typically in the range of tens of megaohms per square. Consequently, they facilitate the high-level transmission of microwaves and radiofrequency waves. Therefore, this outcome can be extrapolated to all designs of germanium-based coatings combined with dielectric materials. In contrast, a thin layer of silver has a sheet resistance in the order of ohms per square, which is much lower than the impedance of a vacuum. This results in significant reflectance of microwaves and radiofrequency waves.

## 4. Conclusions

Decorative coatings made of germanium as a light-absorbing material enable radiofrequency transmission due to their low electrical conductivity in contrast to that of other metallic materials. The deposited thin films of germanium were characterized, resulting in an amorphous structure, a bandgap energy of 0.82 eV, and typical complex refractive indices of amorphous germanium. Various coatings made of germanium and silicon/aluminum nitride have been developed, yielding coatings with a metallic gray appearance using two- and four-layer structures, where the four-layer structure exhibits more reflectance as well as constant transmittance across all the wavelengths. Additionally, black coatings comprising a five-layer structure have been developed, allowing for the adjustment of the coating’s transmittance level. This five-layer structure is also useful for achieving semi-transparent coatings with different colors, such as blue and yellow. The radiofrequency attenuation measurements demonstrate that these types of coatings have a value close to 0 dB, while the other metallic coatings can attenuate up to 30 dB. These coatings could be employed in building glazings without affecting mobile coverage or enabling the use of radar security systems concealed behind decorative metallic car parts.

## Figures and Tables

**Table 1:** Multilayer structure of gray metallic coating.

Type	Structure	Thickness
2 layers	Glass/Ge/SiAlN_x_	Glass 4 mm/78 nm/44 nm
4 layers	Glass/Ge/SiAlN_x_/Ge/SiAlN_x_	Glass 4 mm/22 nm/80 nm/19 nm/42 nm

**Table 2:** Optical properties of two-layer and four-layer gray metallic coatings.

Type	T_vis_ (%)	R_vis_ (%)	Lab_Trans_	Lab_Reflec_
2 layers	2.3	31.1	16.6, 10.5, 23.4	62.5, −1.3, 3.3
4 layers	3.5	45.5	22.1, 2.9, −1.8	73.2, −2.5, 2.4

**Table 3:** Multilayer structure of black coatings (glass/SiAlN_x/_Ge/SiAlN_x/_Ge/SiAlN_x_).

Type	Thickness
Black	Glass 4 mm/47 nm/7 nm/12 nm/51 nm/42 nm
Black+	Glass 4 mm/48 nm/7 nm/20 nm/37 nm/43 nm
Black++	Glass 4 mm/65 nm/9 nm/41 nm/13 nm/59 nm

**Table 4:** Optical properties of black coatings.

Type	T_vis_ (%)	R_vis_ (%)	Lab_Trans_	Lab_Reflec_
Black	4.7	4.6	25.4, 7.8, 16.4	25.6, −0.5, 0.7
Black+	11.6	5.5	40.0, 7.7, 24.1	27.9, −1.3, 4.8
Black++	18.6	4.8	49.5, 7.9, 24.7	26.1, 0.9, 0.9

**Table 5:** Multilayer structure of colored coatings.

Type	Structure	Thickness
Blue	Glass/SiAlN_x_/Ge/SiAlN_x_/Ge/SiAlN_x_	Glass 4 mm/93 nm/9 nm/92 nm/13 nm/30 nm
Light Blue	Glass/SiAlN_x_/Ge/SiAlN_x_	Glass 4 mm/78 nm/64 nm/45 nm
Yellow	Glass/SiAlN_x_/Ge/SiAlN_x_/Ge/SiAlN_x_	Glass 4 mm/39 nm/19 nm/120 nm/11 nm/39 nm

**Table 6:** Optical properties of colored coatings.

Type	T_vis_ (%)	R_vis_ (%)	Lab_Trans_	Lab_Reflec_
Blue	11.2	19.1	40.6, 3.5, 8.6	53.3, −3.9, −30.3
Light Blue	4.1	24.4	23.4, 12.1, 27.8	56.9, −1.7, −12.2
Yellow	14.2	38.9	44.3, −17.5, 15.8	67.6, 2.5, 40.3

## Data Availability

The data presented in this study are available on request from the corresponding author.
